# Arsenic Trioxide Therapy During Pregnancy: ATO and Its Metabolites in Maternal Blood and Amniotic Fluid of Acute Promyelocytic Leukemia Patients

**DOI:** 10.3389/fonc.2022.887026

**Published:** 2022-05-12

**Authors:** Meihua Guo, Jian Lv, Xiaotong Chen, Mengliang Wu, Qilei Zhao, Xin Hai

**Affiliations:** ^1^ Department of Pharmacy, First Affiliated Hospital of Harbin Medical University, Harbin, China; ^2^ Department of Hematology, First Affiliated Hospital of Harbin Medical University, Harbin, China; ^3^ Department of Pharmacy, Heilongjiang University of Chinese Medicine, Harbin, China

**Keywords:** acute promyelocytic leukemia, pregnancy, arsenic trioxide, arsenic species, amniotic fluid, arsenical penetration, fetal arsenic exposure

## Abstract

Acute promyelocytic leukemia (APL) is extremely fatal if treatment is delayed. Management of APL in pregnancy is a challenging situation. Arsenic trioxide (ATO) is successfully applied to treat APL. ATO can be transformed into different arsenic species [arsenite (As^III^), monomethylated arsenic (MMA, consists of MMA^III^ and MMA^V^), dimethylated arsenic (DMA, consists of DMA^III^ and DMA^V^), and arsenate (As^V^)], which produce different toxic effects. Investigating the maternal and fetal exposure to arsenic species is critical in terms of assessing maternal and fetal outcomes, choice of optimal treatment, and making decisions for attempting to preserve the obstetrical and fetal wellbeing. In this study, maternal blood and amniotic fluid (AF) from APL patients treated with ATO in pregnancy and blood samples of non-pregnant patients were collected. Concentrations of inorganic arsenic (iAs, iAs = As^III^+As^V^), MMA, and DMA were analyzed by high-performance liquid chromatography–hydride generation–atomic fluorescence spectrometry (HPLC–HG–AFS). The difference in arsenic species of plasma between pregnant patients and non-pregnant patients, distribution of arsenic compounds in AF and maternal plasma, and arsenic penetration into AF were explored. The outcomes of pregnant women treated with ATO and their fetus were analyzed. No significant differences in arsenic concentration, percentage, and methylation index [PMI: primary methylation index (MMA/iAs); SMI: secondary methylation index (DMA/MMA)] between pregnant women and non-pregnant women (*p* > 0.05) were observed. The mean ratios of AF to maternal plasma were as follows: iAs, 2.09; DMA, 1.04; MMA, 0.49; and tAs, 0.98. Abortion rate is higher with the diagnosis at an earlier gestational age, with 0%, 67%, and 100% of pregnancies ending in abortion during the third, second, and first trimester, respectively. The age of the pregnant women, the dose of ATO, and the duration of fetal exposure *in utero* had no influence on fetal outcomes. All APL women achieved complete remission (CR). Collectively, ATO and its metabolites can easily cross the placenta. Levels and distribution of arsenic species in maternal plasma and AF gave evidence that arsenic species had a different ability to penetrate the placenta into AF (iAs > DMA > MMA) and indicated a relatively high fetal exposure to ATO and its metabolites *in utero*. Gestational age at diagnosis was more likely to be closely related to fetal outcomes, but had no effects on mother outcomes.

## Introduction

Acute promyelocytic leukemia (APL) is a special subtype of acute myeloid leukemia. APL is characterized by life-threatening bleeding complications, which is extremely fatal if treatment is delayed. Arsenic trioxide [ATO, arsenite (As^III^) in solution] has been shown to be highly effective for APL by PML/RARα targeting therapy (1, 2). ATO is recommended in the treatment of both relapsed and newly diagnosed patients (3, 4). APL in pregnancy presents extreme challenges to clinicians with currently limited evidence-based information available. Arsenic is known to be toxic. Understanding the link between maternal and fetal exposure to ATO is critical in terms of choice of optimal treatment and making decision for attempting to preserve the obstetrical and fetal wellbeing.

Published data on the reproductive toxicity of arsenic are limited, which are often restricted to animal studies or environmental exposure. Studies in pregnant animals have shown that exposure to arsenic can result in spontaneous abortion, fetal malformations, and birth defects, which are dose- and time-dependent (4–6). With regard to humans, a few studies were conducted in populations exposed to arsenic from environmental contaminants. Chronic exposure to environmental arsenic has been associated with spontaneous abortion, stillbirth, preterm birth, and neonatal death (7, 8). The mechanisms are poorly understood. Several reports have shown that exposure to arsenic during pregnancy can lead to oxidative stress and inflammation in the placenta and anomalous placental vasculogenesis, which affect pregnancy outcomes like preterm delivery (6, 9).

Arsenic can be metabolized from inorganic arsenic [iAs, arsenite (As^III^) and arsenate (As^V^)] to monomethylated arsenic (MMA, consists of MMA^III^ and MMA^V^) and dimethylated arsenic (DMA, consists of DMA^III^ and DMA^V^) by a sequence of reductions and oxidative methylations (10–12). As^III^ and As^V^ undergo interconversion through natural oxidation and reduction by arsenate reductase (10, 11). The toxicities of different arsenic compounds vary and depend on their valency and concentration (10, 13). The gestation period is one of the most vulnerable periods of human development. Therefore, the evaluation of concentrations and the distribution of arsenic species in APL patients treated with ATO in pregnancy are critical to assess maternal and fetal outcomes in particularly challenging situations. However, no report on arsenic species measurement during pregnancy in APL patients is available. Placenta is important to fetus health, which serves as a protective shield between the fetus and harmful substances in maternal body. Although rarely investigated, some studies have demonstrated that arsenic can pass through the human placenta (14, 15), yet *in utero* fetus exposure to arsenic species during pregnancy in APL patients has not been studied.

Amniotic fluid (AF) plays a central role in quantifying the extent of transplacental passage and evaluating the accumulation of a drug in AF, which ultimately provides insight into the *in utero* drug exposition of the fetus. In this study, high-performance liquid chromatography–hydride generation–atomic fluorescence spectrometry (HPLC–HG–AFS) was used to determine the concentrations of iAs (As^III^ and As^V^), MMA (MMA^III^ and MMA^V^), and DMA (DMA^III^ and DMA^V^) in AF and maternal plasma of pregnant APL patients treated with ATO. The distribution of ATO and its metabolites in AF, arsenic species penetration into AF, and the difference in arsenic species of plasma between pregnant patients and non-pregnant patients were explored for the first time. In addition, the safety of ATO in pregnant patients with APL was assessed based on data of this study and previous reports.

## Patients and Methods

### Patients

This single-center, open-label study was approved by the First Affiliated Hospital of Harbin Medical University Ethics Committee. All patients with APL who were treated with ATO monotherapy in pregnancy were included. For comparison, non-pregnant women with APL who were treated with ATO monotherapy were also taken into this study. The subjects were tested only after written informed consent was obtained. Patients with liver or kidney failure were excluded. The follow-up was performed for the pregnant women who agreed.

### Sample Collection and Determination

Blood samples from pregnant and non-pregnant women patients at the time of trough concentration (C_trough_) were collected just before (within 30 min) the start of daily administration when ATO was continuously administered for >7 days. Maternal venous blood samples were also obtained immediately after delivery. As soon as the sample was collected, blood plasma was separated immediately by centrifugation at 4,000 rpm for 5 min at 4°C. If the collection did not interfere with the clinical management, AF samples were collected using a sterile 30-cc needleless syringe after rupture of membranes. All samples were immediately frozen at −80°C until analysis. The analysis of arsenic species in AF and plasma was performed by HPLC–HG–AFS (LC-AFS 6500, Beijing Haiguang Instruments Co., Ltd., China) (11, 16). The concentrations of arsenic compounds were expressed as the concentrations of the arsenic element (As).

### Sample Preparation

AF or plasma sample (240 μl) was mixed with 120 μl of 30% H_2_O_2_, which was thoroughly vortex-mixed and kept at room temperature overnight. The 360-μl sample was prepared with 40 μl of HClO_4_ (20%) for deproteinization, followed by vortex for 60 s. The mixture was then centrifuged at 15,000 rpm for 15 min at 4°C. The supernatant (100 μl) was injected into the HPLC–HG–AFS system for determination.

### Case Review

To better understand how to manage APL with ATO treatment in pregnancy, we searched the PubMed, Web of Science, CNKI (China), and Wanfang Data (China) database (2009–2022) for articles about maternal and fetal outcomes resulting from APL patients with ATO treatment during pregnancy. Information on patient age, APL risk score, gestational age at diagnosis, treatment program, dose of ATO, duration of fetal exposure *in utero* to ATO, therapy outcome of APL, gestational age at delivery/abortion, delivery method, and fetal outcome was reviewed and investigated.

### Statistical Analysis

Data analysis was performed with GraphPad Prism, version 5.0. A *p*-value <0.05 was considered statistically significant.

## Results

### Patients

Eight plasma samples from 3 pregnant patients (P1–P3) and 5 non-pregnant patients (P4–P8) treated with ATO monotherapy were collected on day 8 after ATO administration. At the time of delivery, maternal plasma and AF samples were obtained from 2 pregnant patients (P1 and P2), but not from patient P3. In this study, 3 pregnant patients and 5 non-pregnant patients, aged from 26 to 38 (32 ± 4) years, were given ATO at a dose of 0.16–0.17 mg/kg once daily. ATO infusion was administered at a continuous slow rate (16, 17). The patients were also given transfusions of platelets, fibrinogen, and erythrocyte. No patients discontinued the treatment of ATO during the therapy. After the treatment, all the patients were negative for PML*-RARa;* fusion transcripts and achieved molecular and hematologic complete remission (CR). Clinical characteristics of these patients are presented in **Table 1**. Fetal outcomes of 3 pregnant women are presented in **Table 4**. Patient P1 and P2 did not accept follow-up. Patient P3 had completed her treatment. The treatment since 2016 has been induction with ATO monotherapy followed by 20 consolidation and maintenance cycles with ATO monotherapy (0.16–0.17 mg/day for 28 days). During the whole treatment phase, the patient showed no complication. The baby’s growth and development were normal.

**Table 1:** Baseline characteristics and therapy outcome of patients in this study.

	Pregnant patients			Non-pregnant patients				
	P1	P2	P3	P4	P5	P6	P7	P8
**Age (years)**	29	31	34	26	37	34	38	29
**WBC count (×10^9^/L)**	6.48	9.58	6.32	15.33	1.14	2.60	2.05	0.62
**Hemoglobin (g/L)**	32.00	97.60	102.60	52.00	53.00	70.20	80.28	95.01
**Platelet count (×10^9^/L)**	30.00	66.70	18.07	14.00	44.00	79.00	59.09	130.80
**PT (s)**	13.3	12.90	15.10	13.80	12.70	20.70	12.60	12.10
**APTT (s)**	26.8	28.50	23.30	25.60	28.30	29.50	24.20	25.50
**FIB (g/L)**	0.76	1.56	0.90	1.34	2.73	0.48	0.94	1.89
**TT (s)**	19.7	14.80	16.30	14.80	15.20	21.80	18.60	15.20
**DD (mg/L)**	5.29	9.05	6.69	7.16	8.24	19.87	5.40	8.32
**ALT (U/L)**	20.10	31.00	28.00	58.00	13.00	20.10	62.00	6.60
**AST (U/L)**	19.90	22.00	17.00	45.00	8.00	22.90	41.00	11.70
**GGT (U/L)**	7.40	75.00	43.00	17.00	21.00	18.50	25.00	12.40
**TBIL (µmol/L)**	8.10	9.87	9.06	13.12	13.10	18.30	17.46	10.20
**BUN (mmol/L)**	3.39	4.22	1.87	3.38	3.04	5.32	4.08	3.41
**Creatinine (µmol/L)**	38.2	69.70	50.30	49.60	55.80	69.60	57.10	58.20
**Magnesium (mmol/L)**	0.74	0.90	0.85	0.87	0.82	0.80	0.74	0.78
**Potassium (mmol/L)**	3.53	4.08	3.57	3.72	3.57	4.04	3.79	3.66
***PML-RARA***	Positive	Positive	Positive	Positive	Positive	Positive	Positive	Positive
**Therapy outcome**	CR	CR	CR	CR	CR	CR	CR	CR

PT, prothrombin time; APTT, activated partial thromboplastin time; FIB, fibrinogen; TT, thrombin time; DD, D-Dimer; ALT, alanine transaminase; AST, aspartate aminotransferase; GGT, γ-glutamyl transpeptidase; TBIL, total bilirubin; BUN, blood urea nitrogen; CR, complete remission.

### Separation of Arsenic Species

**Figure 1:**
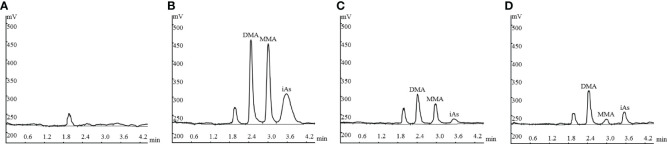
Representative HPLC-HG-AFS chromatograms: drug-free plasma **(A)**; drug-free plasma spiked with standards of arsenic species **(B)**; plasma sample from a patient with APL **(C)**, and amniotic fluid sample from a patient with APL **(D)**. HPLC–HG–AFS: high-performance liquid chromatography–hydride generation–atomic fluorescence spectrometry; iAs: inorganic arsenic; MMA: monomethylarsonic acid; DMA: dimethylarsinic acid.

**Figure 1** shows the representative HPLC–HG–AFS chromatograms of blank plasma, plasma spiked with standard arsenic compounds, plasma sample from a patient with APL, and AF sample from a patient with APL. The DMA, MMA, and iAs in samples and the spiked arsenic standards have the same chromatographic behavior. The interfering peaks from endogenous matrix components were not observed at the retention time.

### Arsenic Species in Plasma of Pregnant and Non-Pregnant Women

Arsenic concentrations of plasma were determined in 8 APL patients treated with ATO. The C_trough_, percentage, and methylation index [PMI: primary methylation index (MMA/iAs); SMI: secondary methylation index (DMA/MMA)] of arsenic species in plasma from pregnant and non-pregnant women are summarized in **Table 2**. The mean arsenic C_trough_ in plasma from pregnant women (*n* = 3) was 17.31 ng/ml for iAs (range: 14.13–19.14 ng/ml), 24.01 ng/ml for DMA (range: 16.89–28.65 ng/ml), 23.42 ng/ml for MMA (range: 13.60–22.42 ng/ml), and 64.74 ng/ml for tAs (total arsenic, tAs = iAs + DMA + MMA) (range: 54.53–78.26 ng/ml). For non-pregnant women (*n* = 5), the mean arsenic C_trough_ in plasma was 16.53 ng/ml for iAs (range: 9.83–20.14 ng/ml), 18.19 ng/ml for DMA (range: 9.19–23.28 ng/ml), 18.41 ng/ml for MMA (range: 15.35–21.84 ng/ml), and 53.13 ng/ml for tAs (range: 44.67–58.87). There were higher arsenical concentrations in the plasma of pregnant women than that of non-pregnant women. However, no significant differences in C_trough_ levels of arsenic species, percentage (iAs%, DMA%, and MMA%), and methylation index (PMI and SMI) between the two sets were observed (*p* > 0.05).

**Table 2:** The C_trough_, percentage, and methylation index of arsenic species in plasma from pregnant and non-pregnant women treated with arsenic trioxide (ATO), mean (range).

		iAs	DMA	MMA	tAs	PMI	SMI
**Pregnant patients (*n* = 3)**	**C_trough_** (ng/ml)	17.31 (14.13–19.14)	24.01 (16.89–28.65)	23.4 2 (13.60–22.42)	64.74 (54.53–78.26)		
	**Percentage** (%)	26.74 (23.00–34.21)	37.08 (30.98–46.64)	36.18 (30.36–41.71)			
	**Methylation index**					1.35 (1.02–1.71)	1.02 (0.81–1.54)
**Non-pregnant patients (*n* = 5)**	**C_trough_** (ng/ml)	16.53 (9.83–20.14)	18.19 (9.19–23.28)	18.41 (15.35–21.84)	53.13 (44.67–58.87)		
	**Percentage** (%)	31.11 (19.38–45.08)	34.24 (20.57–45.9)	34.65 (26.58–40.86)			
	**Methylation index**					1.11 (0.76–1.79)	0.99 (0.60–1.71)

C_trough_, trough concentration; iAs, inorganic arsenic; MMA, monomethylarsonic acid; DMA, dimethylarsinic acid; tAs, total arsenic, tAs = iAs + DMA + MMA. Percentage (%). (Concentrations of arsenic species/Concentration of tAs) × 100%. PMI, primary methylation index (MMA/iAs); SMI, secondary methylation index (DMA/MMA).

### Comparisons of Arsenic Species in AF With That in Maternal Blood

Arsenical concentrations in maternal plasma and AF of 2 pregnant patients at the time of delivery (4–6 days after the end of ATO treatment) were determined. The arsenic concentration of AF/maternal plasma ratio was calculated to reflect the AF penetration efficiency for arsenic compounds. The arsenic species concentrations, percentage, and methylation index in AF and maternal plasma and penetrations (AF concentration/maternal plasma concentration) in AF of pregnant patients are shown in **Table 3**. For the 2 pregnant patients, the mean iAs, DMA, MMA, and tAs concentrations of AF were 5.71 ng/ml, 8.98 ng/ml, 3.57 ng/ml, and 18.25 ng/ml, respectively. The overall concentration distribution trend in AF of the patients was DMA > iAs > MMA. The mean iAs, DMA, MMA, and tAs concentrations of AF/maternal plasma ratios were 2.09, 1.04, 0.49, and 0.98 after the end of induction therapy, respectively. The higher iAs levels in AF than that in maternal plasma from pregnant patients treated with ATO were observed. The iAs and DMA tended to exhibit higher penetrations into AF than MMA. The overall trend of penetration into AF of arsenic species in the 2 patients was iAs > DMA > MMA, which was different from the trend of DMA > MMA > iAs in maternal plasma.

**Table 3:** Arsenic species in amniotic fluid (AF) and maternal plasma of APL patients treated with arsenic trioxide in pregnancy.

Patients	Sample time		Concentration (ng/ml)		Ratio(C_AF_/C_MP_);
			Maternal plasma (C_MP_)	Amniotic fluid (C_AF_)	
P1	4 days after the end of induction therapy	iAs	3.56	5.81	1.63
		DMA	9.29	11.71	1.26
		MMA	8.07	4.79	0.59
		tAs	20.92	22.31	1.07
		PMI	2.27	0.82	/
		SMI	1.15	2.44	/
		iAs%	17.02	26.04	/
		DMA%	44.41	52.49	/
		MMA%	38.58	21.47	/
P2	6 days after the end of induction therapy	iAs	2.20	5.60	2.55
		DMA	7.68	6.25	0.81
		MMA	6.15	2.34	0.38
		tAs	16.03	14.19	0.89
		PMI	2.80	0.42	/
		SMI	1.25	2.67	/
		iAs%	13.72	39.46	/
		DMA%	47.91	44.05	/
		MMA%	38.37	16.49	/

C_AF_/C_MP_, AF concentration/maternal plasma concentration; iAs, inorganic arsenic; MMA, monomethylarsonic acid; DMA, dimethylarsinic acid; tAs, total arsenic, tAs = iAs + DMA + MMA. Percentage (%). (Concentrations of arsenic species/Concentration of tAs) × 100%. PMI, primary methylation index (MMA/iAs); SMI, secondary methylation index (DMA/MMA).

### Case Review

We performed a systematic retrospective review to analyze the outcomes reported for both mother and fetus when APL is diagnosed and treated with ATO during pregnancy. Literature databases were systematically searched to identify studies reporting cases of ATO treatment during pregnancy. Eighteen published articles met the eligibility criteria. The extracted data from the selected articles are presented in **Table 4**. A total of 28 APL patients from literature were eligible for ATO therapy in pregnancy, with most of them being treated with ATO plus all-trans retinoic acid (ATRA) (*n* = 12, 43%) or ATO plus ATRA combined with chemotherapy (*n* = 15, 54%), while the remaining patients received ATO monotherapy (*n* = 1, 14%). All patients achieved CR during pregnancy or after delivery (100%). The age of women (*n* = 18) who experienced spontaneous or induced abortion was 27.8 ± 2.8 years, which was not significantly different from the age (28.0 ± 4.7 years) of women (*n* = 10) who delivered normal babies. The mean dose of ATO and duration of fetal exposure *in utero* to ATO of women who experience spontaneous or induced abortion were lower than that of women who delivered normal babies, 258 mg (*n* = 5) vs. 398 mg (*n* = 5) and 31.6 days (*n* = 17) vs. 40.2 days (*n* = 9), respectively. Evidently, apart from mother age, fetal viability is not related to dose of ATO and duration of fetal exposure *in utero* to ATO.

**Table 4:** Clinical characteristics and maternal and fetal outcomes in pregnant women with APL treated with ATO in this study and literature.

	Case no.	Age, years	APL risk score	Gestational age at diagnosis, weeks	Treatment	Dose of ATO received	Fetal exposure *in utero* to ATO, days	Therapy outcome	Gestational age at delivery/abortion, weeks	Delivery method	Fetal outcome
Cases in this Study	1	29	Intermediate	17^+5^	ATO monotherapy	0.17 mg/kg/day (10 mg/day)	28	CR	23^+4^	TA (polyhydramnios)	Aborted fetus
	2	31	Low	20	ATO monotherapy	0.16 mg/kg/day (10 mg/day)	28	CR	24	TA	Aborted fetus
	3	34	Low	37	ATO monotherapy	0.16 mg/kg/day (10 mg/day)	15	CR	39^+1^	Vaginal	Healthy infant
Cases in literature	1 (18)	30	\	2	ATO+ATRA+DNR	\	57	CR	10+1	TA	Aborted fetus
	2 (19)	25	Low/Intermediate	5	ATO+ATRA+DNR	\	28	CR	9	SA	Aborted fetus
	3 (19)	28	Low/Intermediate	5	ATO+ATRA+IDA	\	14	CR	7	TA	Aborted fetus
	4 (20)	25	High	8	ATO+ATRA+DNR	\	21	CR	11	TA	Aborted fetus
	5 (21)	32	Intermediate	9	ATO+ATRA	10 mg/day	21	CR	12	TA	Aborted fetus
	6 (22)	25	Low	9^+5^	ATO+ATRA	10 mg/day	23	CR	13^+1^	TA	Aborted fetus
	7 (23)	26	High	10	ATO+ATRA+DNR	\	21	CR	13	TA	Aborted fetus
	8 (19)	26	Low/Intermediate	10	ATO+ATRA	\	35	CR	15	TA	Aborted fetus
	9 (24)	25	Low	11^+3^	ATO+ATRA	0.15 mg/kg	14	CR	14^+1^	TA	Aborted fetus
	10 (18)	31	\	12	ATO+ATRA+DNR	\	80	CR	23^+3^	TA	Aborted fetus
	11 (25)	33	Low	12^+4^	ATO+ATRA+IDA	10 mg/day	35	CR	17^+4^	TA	Aborted fetus
	12 (18)	23	\	12^+4^	ATO+ATRA+DNR	\	72	CR	23^+6^	TA	Aborted fetus
	13 (19)	27	Low/Intermediate	13	ATO+ATRA+DNR	\	28	CR	17	TA	Aborted fetus
	14 (26)	22	Intermediate	14	ATO+ATRA	0.15 mg/kg/day (10 mg/day)	93	CR	40^+6^	Vaginal	Healthy infant
	15 (27)	28	Intermediate	23^+4^	ATO+ATRA	\	25	CR	28	TA	Aborted fetus
	16 (24)	28	Intermediate	24^+4^	ATO+ATRA	0.15 mg/kg	23	CR	29^+4^	TA	Aborted fetus
	17 (22)	28	Intermediate	24^+5^	ATO+ATRA	10 mg/day	30	CR	29	TA	Aborted fetus
	18 (28)	23	High	27	ATO+ATRA +IDA	0.15 mg/kg	35	CR	32	Cesarean	Healthy infant
	19 (29)	29	\	27	ATO + ATRA + DNR	\	\	CR	\	TA	Aborted fetus
	20 (30)	31	Intermediate	27	ATO monotherapy	10 mg/day	10	CR	29	ID+TA	Aborted fetus
	21 (30)	30	Intermediate	27^+1^	ATO+ATRA+HHRT	10 mg/day	70	CR	37	Cesarean	Healthy infant
	22 (18)	33	\	28	ATO+ATRA	\	87	CR	40^+3^	Vaginal	Healthy infant
	23 (31)	27	Intermediate	31^+^	ATO+ATRA+DNR	10 mg/day	14	CR	33^+^	Cesarean (fetal distress)	Healthy infant
	24 (32)	37	\	32	ATO+ATRA	\	20	CR	\	Cesarean	Healthy infant
	25 (33)	28	Intermediate	34^+4^	ATO+ATRA+DNR	10 mg/day	16	CR	37^+5^	Cesarean	Healthy infant
	26 (34)	23	Low	35	ATO+ATRA	0.15 mg/kg	21	CR	38	Vaginal	Healthy infant
	27 (35)	27	Low	36	ATO+ATRA	10 mg/day	6	CR	38	Cesarean	Healthy infant
	28 (29)	30	\	36	ATO+ATRA+DNR	\	\	CR	\	Cesarean	Healthy infant

APL, acute promyelocytic leukemia; CR, complete remission; ATO, arsenic trioxide; ATRA, all-trans-retinoic acid; TA, therapeutic abortion; SA, spontaneous abortion; DNR, daunorubicin; IDA, idarubicin; HHRT, homoharringtonine; ID, Intrauterine death.

For the pregnant women diagnosed with APL in the first trimester, all of them (*n* = 10, 100%) experienced spontaneous or induced abortion. For the ones diagnosed with APL during the second trimester, 8 of 12 (67%) patients’ gestation ended in abortion. The remaining 4 (33%) women who were diagnosed with APL during the second trimester continued gestation until delivery of healthy infants by vaginal delivery or cesarean section. For the APL patients during the third trimester, all pregnancies (*n* = 6, 100%) delivered normal babies. APL patients treated with ATO during the first trimester were more susceptible to spontaneous and induced abortion compared with those during the second trimester and the third trimester (100% vs. 67% and 100% vs. 0%) (*p* < 0.0001). Thus, gestational age at diagnosis was instead crucial in fetal outcomes. In these cases, an interesting case should be paid attention to (26). A 22-year-old woman was diagnosed with APL at only 14 weeks of gestation. The fetus received a total exposure to ATO of 93 days and 930 mg. A healthy infant was delivered at 40^6+^ weeks, and the mother achieved CR.

For the 3 patients in this study, information on APL risk score, gestational age at diagnosis, treatment program, dose of ATO, duration of fetal exposure *in utero* to ATO, therapy outcome of APL, gestational age at delivery/abortion, delivery method, and fetal outcome was also investigated. As presented in **Table 4**, 3 patients received ATO monotherapy (100%), which is different from that in previous reports. The gestation of 2 pregnant women diagnosed with APL during the second trimester ended in abortion. The APL patient during the third trimester delivered a normal baby. The fetal outcomes in the 3 cases seemed to be in accord with the above characteristics; that is to say, gestational age at diagnosis was instead crucial in fetal outcomes.

## Discussion

ATO has been successfully used for front-line treatment of APL (36). As a well-known poison, ATO treatment for APL during pregnancy is a significant challenge, which is associated with emergency treatment for APL, fetal exposure to arsenic, and pregnancy outcomes. Clinical trial is obviously impossible and no studies are available regarding the use of ATO in pregnant women. The only data available are from animal or environmental exposure studies. The current study is the first study to investigate the ATO and its metabolite concentrations in the plasma and AF from APL patients treated with ATO during pregnancy, which reflects the fetal exposure levels to arsenic compounds during ATO treatment and the penetration efficiency of arsenic species into AF in the real world. The dose in these cases is much higher than in environmental studies, which is crucial to clinical treatments. In addition, the clinical cases on the application of ATO use during pregnancy in the literature were systematically searched and analyzed in this study. Our results may help medical teams make hard decisions in extremely complex clinical situations such as APL treatment during pregnancy.

Published reports and our preliminary results suggested that ATO is biotransformed into two types of major metabolites through oxidative methylation in human: MMA and DMA, which is catalyzed by arsenic methyltransferase (10, 11, 16, 17). As^V^ is a rare metabolite in the process (16). The prototype drug As^III^ and its metabolites are widely distributed throughout the body. The arsenic species can be detected in urine (11), plasma (16), red blood cells (17), leukocytes (37), granulocytes (37), and cerebrospinal fluid (CSF) (38) of APL patients treated with ATO, and in kidney (39), liver (39), and heart (39) of rat treated with ATO. In this study, the C_trough_, percentage, and methylation index of arsenic species in plasma from pregnant and non-pregnant women treated with ATO were evaluated. As shown in **Table 2**, no significant differences between the two sets were observed. The reasons may be the small sample size or no significant influence of pregnancy on arsenic metabolism.

Another interesting finding of this study was the remarkable differences in the arsenic species levels in the plasma from those in our preliminary published report (16). Higher levels of DMA, iAs, MMA, and tAs were measured in the present study. The discrepancy might have resulted from the different sample preparation methods between the two studies. H_2_O_2_ was added to plasma during sample preparation to cleave the bonds between arsenic and plasma proteins. In this process, trivalent arsenicals (As^III^, DMA^III^, and MMA^III^) were oxidized to pentavalent arsenicals (As^v^, DMA^v^, and MMA^v^) by H_2_O_2_ (40). Based on our study (17) and previous reports (40), H_2_O_2_ at this concentration changed the oxidation state of arsenicals, but not the methylation status. Therefore, the detected arsenic species were the sum of unbound arsenicals and protein-bound arsenicals that were released and oxidized by H_2_O_2_.

Our published study showed that As^III^ and its metabolites have a limited ability to penetrate the blood–brain barrier into CSF (38). Arsenical concentration in CFS is much lower than that in plasma (38). In this study, our results showed that arsenic concentration in AF is much higher than that in maternal plasma, which suggested that As^III^ and its metabolites have a strong ability to penetrate the placental barrier into AF.

The overall concentration trend of arsenic species in AF of in the 2 APL patients was DMA > iAs > MMA during the drug-withdrawal period, which was different from that in maternal plasma (DMA > MMA > iAs). Our preliminary results indicated that the overall penetration into CSF of arsenic species was iAs > DMA^V^ > MMA^V^ (As^V^ was not detected in CSF) (38). It is interesting that a similar thing was observed in this study. Our results showed that the penetration into AF of iAs was higher than DMA and MMA, which suggested that the iAs may possess stronger placental barrier permeability and the MMA exhibited weaker placental barrier penetration efficiency. The results also implied higher iAs accumulation in the AF compared with DMA and MMA. Some reasons for this phenomenon can be traced as follows: (1) At middle and late gestation, fetal urine is produced, contributing to most of the volume and composition of the AF. DMA^V^ and As^III^ were the dominant arsenic compounds excreted from the urine (11). (2) Unbound arsenicals possess stronger barrier permeability than protein-bound arsenicals. The different placental barrier permeability of arsenic species probably comes from the different chemical structure or protein binding capacity. (3) The arsenic ingested by fetus could undergo fetal clearance by its metabolic pathways. Because of the lower metabolism of fetus, there is much higher level of un-metabolized As^III^ in AF than in maternal plasma. (4) There may be an increased penetration into and/or accumulation within AF or a decreased elimination out of AF. The exact reason is unknown and further explorations are needed.

Taken together, these data from our study indicated that maternally treated ATO (As^III^) and its pharmacologically metabolites can easily pass through the placental barrier. Thus, *in utero*, the fetus could be simultaneously exposed to ATO and its metabolites by fetus swallowing AF. It has become clear that arsenic metabolites also possess cytotoxicity (13, 41). Previous studies have shown that trivalent methylated arsenicals (MMA^III^ and DMA^III^) are much more toxic than iAs (13, 42). The concentration trend of DMA > iAs > MMA in AF suggests a higher fetal exposure to DMA. The higher permeability of the placental barrier and the lower affinity of fetal plasma proteins to the drugs could increase fetal exposure to the circulating drug and its metabolites. Our results also showed that there was a higher iAs level and a lower MMA level in the AF than those in the maternal plasma. Furthermore, our data suggest that continuous fetal exposure to ATO and its metabolites *via* re-circulation of the AF could occur. The biological consequences of fetal exposure to maternally administered ATO and/or its metabolites *via* placental transfer and re-circulation of AF are yet to be determined. Further studies are needed to evaluate the effect(s) of iAs, DMA, and MMA on the neonatal outcome of infants exposed to the drug and its metabolites *in utero*.

The diagnosis and management of APL in pregnancy presents significant challenges. It is impossible to prospectively study the appropriate measures for the management of pregnant women with APL. A systematic review of the literature cases seems the best way to obtain evidence-based information to guide decision-making in clinical practice. To analyze the maternal and fetal outcomes of APL patients treated with ATO in pregnancy, a systematic literature review was performed for the first time in this study. The results suggested that all pregnant women with APL (*n* = 28) in literature reports achieved CR (CR rate was 100%). The correlation between fetal outcomes and age, dose of ATO, and duration of fetal exposure *in utero* to ATO was not observed. There was an abortion rate of 100%, 67%, and 0% in those diagnosed with APL during the first, second, or third trimester of pregnancy in 28 cases included, respectively. The results suggested that the chances of achieving CR for the pregnant women with APL remained very high by ATO treatment, regardless of gestational age at diagnosis. In other words, for the mother, gestational age has no significant influence on the probability of achieving CR. In contrast, fetal outcome is strongly related to gestational age. In these cases, polyhydramnios, jaundice, respiratory distress syndrome, and intrauterine death were observed during the perinatal period. However, teratogenic effects were not observed. Our results indicated that ATO and its metabolites can penetrate the placenta into AF. The concentration ratios of AF to maternal plasma highlighted a relatively high fetal exposure to arsenic compounds. Based on overall consideration, although teratogenic effects were not observed in this study, avoiding ATO during the early stage of pregnancy is recommended, due to its potential teratogenicity and other toxicity. Some reports support the proposal that the treatment of APL in pregnancy should give priority to an anthracycline, particularly daunorubicin (43, 44). More data and further exploration are needed to testify this. Comparing ATO treatment with daunorubicin or anthracycline treatment in a large sample is recommended. Our result suggested that ATO and chemotherapy seem to be reasonably safe when given to APL patients during the third trimester of pregnancy. Some reports indicated successful pregnancies after the end of ATO therapy for APL (4, 44–46). Even so, to avoid a fetal exposure to ATO, using this agent only after delivery is recommended.

Our study had some limitations. Acute leukemia in pregnancy has a low incidence (1 out of 100,000 pregnancies) (26). APL is a variant of acute myeloid leukemia (AML) with an incidence of approximately 2–3 per million. Then, APL during pregnancy is rather rare. In addition, obtaining AF is a prominent difficulty. Therefore, the small number of patients and AF samples included in our study poses a considerable shortcoming. Our findings do not allow for general conclusions on arsenic species concentrations in AF and maternal circulation within APL patients treated with ATO during pregnancy. Given that AF determinations were performed during delivery when ATO was withdrawn, drug concentrations of AF do not represent C_trough_ (trough levels), thus limiting the generalizability of the results. In our previous report (37), arsenic species could still be detected in blood cells at the time of drug withdrawal for 3–109 days. In this study, we collected the maternal blood samples and AF samples from 2 APL patients at 4 and 6 days after the treatment. Unfortunately, the samples from later time points were also not obtained due to the lack of clinical compliance or informed consent. Thus, arsenic metabolism and clearance after treatment in AF or maternal blood were not evaluated. Umbilical cord blood were not obtained. The conclusions on the correlation between maternal plasma concentration of arsenic species and cord blood levels were not made at this time. Besides that, further information about the long-term safety of arsenic compounds in terms of pregnancy outcomes and effects on fetus was lacking in our investigation. Further studies and follow-up are required.

Despite the above limitations, data from our *in vivo* study demonstrated the characteristics of intrauterine arsenic exposure and the permeability of placenta for ATO and its metabolites for the first time. The first safety assessment of ATO in pregnant women with APL was performed in this study. Our studies will enable further analysis of the possible effects of ATO and its metabolites on maternal and fetal outcomes.

## Conclusions

In summary, ATO and its metabolites in AF and maternal plasma of pregnant APL patients treated with ATO were measured by HPLC–HG–AFS for the first time. There were no significant differences in C_trough_ levels of arsenic species, percentage (iAs%, DMA%, and MMA%), and methylation index (PMI and SMI) between pregnant women (*n* = 3) and non-pregnant women (*n* = 5) (*p* > 0.05), which may be due to the small size or no significant influence of pregnancy on arsenic metabolism. The overall concentration distribution trend in AF was DMA > iAs > MMA, which was different from that in the corresponding maternal plasma, DMA > MMA > iAs. These results suggested that arsenic compounds (iAs, DMA, and MMA) had the ability to easily cross the human placenta barrier and appear in AF, fetuses were exposed to relatively high levels of ATO and its metabolites *in utero* during maternal ATO treatment, and arsenic species exhibited different penetrations into AF (iAs> DMA > MMA); in other words, iAs possessed stronger placental barrier permeability and the MMA exhibited weaker placental barrier penetration efficiency, which was similar to that of arsenic penetration into CSF.

A systematic literature review about APL women in pregnancy treated with ATO was performed. Combined with the data of this study, the results indicated that mother age, the dose of ATO, and duration of fetal exposure *in utero* had no influences on fetal outcomes. Gestational age was closely related to fetal outcomes, but did not affect mother outcomes (CR rate, 100%). In brief, avoiding ATO treatment during the early stage of pregnancy should be emphasized, due to high fetal exposure to ATO and its metabolites *in utero*, ATO potential teratogenicity, and other toxicity.

These results may be beneficial for medical teams to assess maternal and fetal outcomes, preserve the obstetrical and fetal wellbeing, and make hard decisions such as ATO treatment for pregnant women with APL. Since this is the first report that analyzed the arsenical concentrations in maternal blood and AF and evaluated the permeability of the placenta barrier for arsenic species in pregnant APL patients receiving ATO, these findings need to be further investigated and should be corroborated using a larger sample population in multi-center studies.

## Data Availability Statement

The original contributions presented in the study are included in the article/supplementary material. Further inquiries can be directed to the corresponding author.

## Ethics Statement

The studies involving human participants were reviewed and approved by the Ethics Committee of the First Affiliated Hospital of Harbin Medical University. The patients/participants provided their written informed consent to participate in this study.

## Author Contributions

MG and JL carried out experimental work, analyzed the data, and wrote the manuscript. XC collected the samples. MG, MW, and QZ contributed to the final preparation of this paper and submission. XH and XC revised the manuscript. XH designed and supervised this research. All authors contributed to the article and approved the submitted version.

## Funding

This study was supported by the National Natural Science Foundation of China (No. 81700151), the Natural Science Foundation of Heilongjiang Province for Excellent Youths (No. YQ2019H016), the Excellent Youth Foundation of First Affiliated Hospital of Harbin Medical University (No. HYD2020JQ0018), the Heilongjiang Key R&D Program (No. GZ20210070), the Heilongjiang Postdoctoral Program (No. LBH-Q20031), and the Foundation of First Affiliated Hospital of Harbin Medical University (No. 2019M15).

## Conflict of Interest

The authors declare that the research was conducted in the absence of any commercial or financial relationships that could be construed as a potential conflict of interest.

## Publisher’s Note

All claims expressed in this article are solely those of the authors and do not necessarily represent those of their affiliated organizations, or those of the publisher, the editors and the reviewers. Any product that may be evaluated in this article, or claim that may be made by its manufacturer, is not guaranteed or endorsed by the publisher.
